# Optimization of GaAs Nanowire Pin Junction Array Solar Cells by Using AlGaAs/GaAs Heterojunctions

**DOI:** 10.1186/s11671-018-2503-8

**Published:** 2018-04-25

**Authors:** Yao Wu, Xin Yan, Wei Wei, Jinnan Zhang, Xia Zhang, Xiaomin Ren

**Affiliations:** 1 State Key Laboratory of Information Photonics and Optical Communications, Beijing University of Posts and Telecommunications, Beijing, 100876 China; 2 School of Mechanical and Electric Engineering, Guangzhou University, Guangzhou, 510006 China

**Keywords:** Nanowire array, Photovoltaic, Heterojunction, GaAs nanowire

## Abstract

We optimized the performance of GaAs nanowire pin junction array solar cells by introducing AlGaAs/GaAs heterejunctions. AlGaAs is used for the p type top segment for axial junctions and the p type outer shell for radial junctions. The AlGaAs not only serves as passivation layers for GaAs nanowires but also confines the optical generation in the active regions, reducing the recombination loss in heavily doped regions and the minority carrier recombination at the top contact. The results show that the conversion efficiency of GaAs nanowires can be greatly enhanced by using AlGaAs for the p segment instead of GaAs. A maximum efficiency enhancement of 8.42% has been achieved in this study. And for axial nanowire, by using AlGaAs for the top p segment, a relatively long top segment can be employed without degenerating device performance, which could facilitate the fabrication and contacting of nanowire array solar cells. While for radial nanowires, AlGaAs/GaAs nanowires show better tolerance to p-shell thickness and surface condition.

## Background

GaAs nanowires (NWs) have been considered as potential building blocks for high efficiency solar cells [1–3]. With a bandgap of 1.43 eV, GaAs is more favorable than Si for maximizing the efficiency of solar cells [4]. An efficiency of 15.3% has been achieved by a GaAs NW array with axial pn junctions [5]. However, due to the fact that GaAs NW solar cells always suffer from serious surface recombination, surface passivation is necessary for achieving satisfactory performance [6, 7]. A common method for GaAs NW passivation is to form an AlGaAs shell around the NW, which creates large barriers for both electrons and holes throughout the structure, preventing the minority carriers from being recombined at the surface [5, 8, 9].

Except for surface passivation, enhancing light absorption in the active regions is also an effective method to improve the conversion efficiency, which facilitates the electron–hole separation. For NW solar cells with pn junctions, the optimized efficiency can be achieved by placing the junction near the position where the most carriers are generated [10–12], while for pin junction solar cells, higher efficiency can be achieved if more carriers can be generated in the intrinsic regions [13–17]. What is more, by suppressing the optical generation in regions near the contacts, the number of photogenerated minority carriers that diffuse into the contacts can be decreased [14, 17]. There are many methods to enhance light absorption in active regions, such as adjusting junction positions or lengths [13, 14], employing inclined NWs [15], decorating the active region with metal particles [16], or fabricating the heavily doped regions with high bandgap materials [17]. For GaAs NW solar cells, the usage of AlGaAs shells as passivation layers has been widely reported. However, the ability of AlGaAs/GaAs heterostructures to confine photogenerated carriers in the active regions has been paid less attention.

In this paper, we optimized the performance of GaAs NW pin junction array solar cells by employing AlGaAs/GaAs heterojunctions. Both axial and radial junctions have been investigated. In the AlGaAs/GaAs pin heterojunction structures, AlGaAs is used for the p type top segment for axial junctions and the p type outer shell for radial junctions. Due to the relatively low absorption coefficient of AlGaAs, fewer photocarriers are generated in the p-regions. Consequently, more photocarriers are concentrated in the i-regions. Therefore, the recombination loss caused by high doping concentration can be suppressed. Moreover, the high bandgap AlGaAs layers can effectively deflect minority carriers away from the NW surfaces or contacts to decrease minority carrier recombination.

The AlGaAs/GaAs pin heterojunction NW array solar cells have been investigated by a coupled three-dimensional (3-D) optoelectronic simulation, and their performance has been compared with GaAs NW arrays with the same geometry structures. The results show that, by using AlGaAs for the p segment instead of GaAs, the efficiency of axial junction solar cells can be improved even with long top p segments, while for radial junction solar cells, the efficiency can be maintained at a relatively high value with very high surface recombination velocities (SRVs).

## Methods

**Fig. 1:**
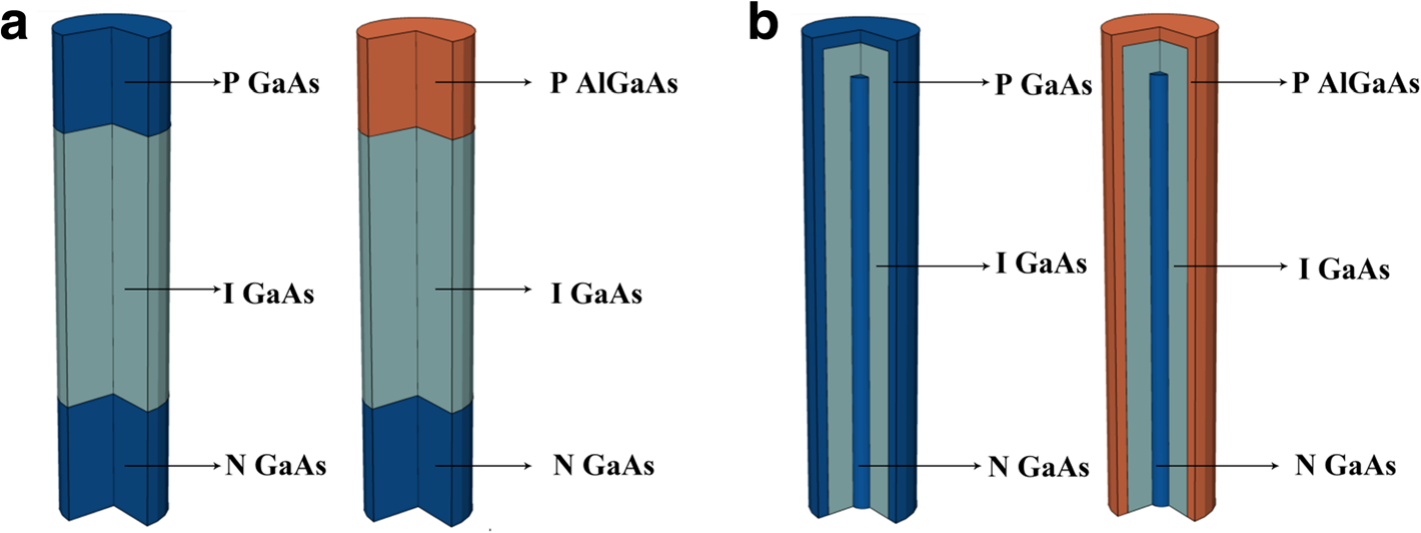
**a** The schematic drawings of the GaAs nanowire axial pin junction solar cell and its AlGaAs/GaAs heterojunction counterpart. **b** The schematic drawings of the GaAs nanowire radial pin junction solar cell and its AlGaAs/GaAs heterojunction counterpart

The schematic of the GaAs nanowire pin junction array solar cell and its AlGaAs/GaAs heterojunction counterparts are illustrated in Fig. 1; each solar cell contains a periodic NW array, of which only a single NW is shown. To fabricate AlGaAs/GaAs heterojunctions, Al_0.8_Ga_0.2_As is used for the top p type segment for axial pin junctions and the outer p type shell for radial pin junctions; the other regions of the NWs are made of GaAs. The doping concentration of both p and n regions is 10^18^ cm^− 3^. The NW diameter and length are 180 nm and 1.2 μm, and the array period is 360 nm; these geometry parameters are chosen according to [18], where the light absorption of GaAs NW arrays has been optimized by adjusting the D/P ratio and NW diameter.

For optical calculation, we use the software package FDTD Solutions (Lumerical, Inc.) to calculate the absorption profile in the NWs. By placing periodic boundary conditions, the simulations can be carried out using a single NW to model the periodic array structure. The complex refractive index of GaAs and Al_0.8_Ga_0.2_As used in the simulation are taken from [19]. The number of absorbed photons at each grid point is calculated from the Poynting vector S, assuming that each photon absorbed generates one electron–hole pair:

1 $$ {G}_{ph}=\frac{\left|\overrightarrow{\nabla}\cdot \overrightarrow{S}\right|}{2\mathrm{\hslash}\omega }=\frac{\varepsilon^{{\prime\prime} }{\left|\overrightarrow{E}\right|}^2}{2\mathrm{\hslash}} $$

where ℏ is the reduced Planck’s constant, *ω* is the angular frequency of the incident light, *E* is the electric field intensity at each grid point, and *ε*″ is the imaginary part of the permittivity. To obtain the optical generation rate profile used for electric simulation, *G*_*ph*_ is weighted by the AM 1.5G solar spectrum and integrated over the simulation spectrum.

For the electrical modeling, the optical generation profiles are incorporated into the finite-element mesh of the NWs using Synopsys Sentaurus, which solves the carrier continuity equations coupled with Poisson’s equation self-consistently. The doping-dependent mobility, radiative, Auger, and Shockley-Reed-Hall (SRH) recombination are taken into consideration in the device electrical simulation. The heterojunction between AlGaAs and GaAs is modeled using the thermionic emission model [20]. The electron and hole currents (*J*_*n*_ and *J*_*p*_) across the heterostructure can be described as:

2 $$ {J}_n={a}_nq\left[{v}_{n,2}{n}_2-\frac{m_{n,2}}{m_{n,1}}{v}_{n,1}{n}_1\exp \left(-\frac{\varDelta {E}_c}{k_BT}\right)\right] $$

3 $$ {J}_p=-{a}_pq\left[{v}_{p,2}{p}_2-\frac{m_{p,2}}{m_{p,1}}{v}_{p,1}{p}_1\exp \left(-\frac{\varDelta {E}_v}{k_BT}\right)\right] $$

where *a*_*n*_(*a*_*p*_) is the thermionic current coefficients, *q* is the elementary charge, *v*_*n*_(*v*_*p*_) is the emission velocity of the electrons (holes), which can be expressed as follows:

4 $$ {v}_n=\sqrt{k_BT/2\pi {m}_n} $$

5 $$ {v}_p=\sqrt{k_BT/2\pi {m}_p} $$

and *n*(*p*) is the electron(hole) density, and m_n_(m_p_) is the effective mass of the electrons(holes). *k*_*B*_ is the Boltzmann constant, and *T* is the temperature set to be room temperature in the simulation. The subscripts 1 and 2 represent the materials with the lower and higher conduction band edges, respectively. Δ*E*_*c*_ and Δ*E*_*v*_ are the conductive and valence band offsets at the GaAs/AlGaAs interface. We assume that the interface between AlGaAs and GaAs is perfect without any additional recombination centers. This is usually valid for the lattice-matched epitaxy of AlGaAs on GaAs [21]. Surface recombination is only considered for the interfaces between air and NWs. The parameters used in the device simulation are listed in Table 1. The Auger coefficients, radiative recombination coefficients, and SRH recombination lifetimes of AlGaAs and GaAs are set to be the same [11, 12].

**Table 1:** Key simulation parameters

Parameters	Values (GaAs)	Values (AlGaAs)
Electron and hole mobility	Doping dependent	Doping dependent
Bandgap	1.43 eV	2.1 eV
Electron relative effective mass	0.067m_0_	0.115m_0_
Hole relative effective mass	0.485m_0_	0.598m_0_
Thermionic current coefficients	2	2
SRH lifetimes for electrons and holes	1 ns	1 ns
Radiative recombination coefficient	7.2 × 10^−10^ cm^3^/s	7.2 × 10^−10^ cm^3^/s
Auger recombination coefficient	1 × 10^−30^ cm^6^/s	1 × 10^−30^ cm^6^/s
Surface recombination velocity	10^3^ cm/s or 10^7^ cm/s	10^3^ cm/s or 10^7^ cm/s
Recombination velocity at contacts	10^7^ cm/s	10^7^ cm/s
Conduction band offset at GaAs/Al_0.8_Ga_0.2_As interface	0.315 eV	
Valence band offset at GaAs/Al_0.8_Ga_0.2_As interface	0.31 eV	

## Results and Discussion

**Fig. 2:**
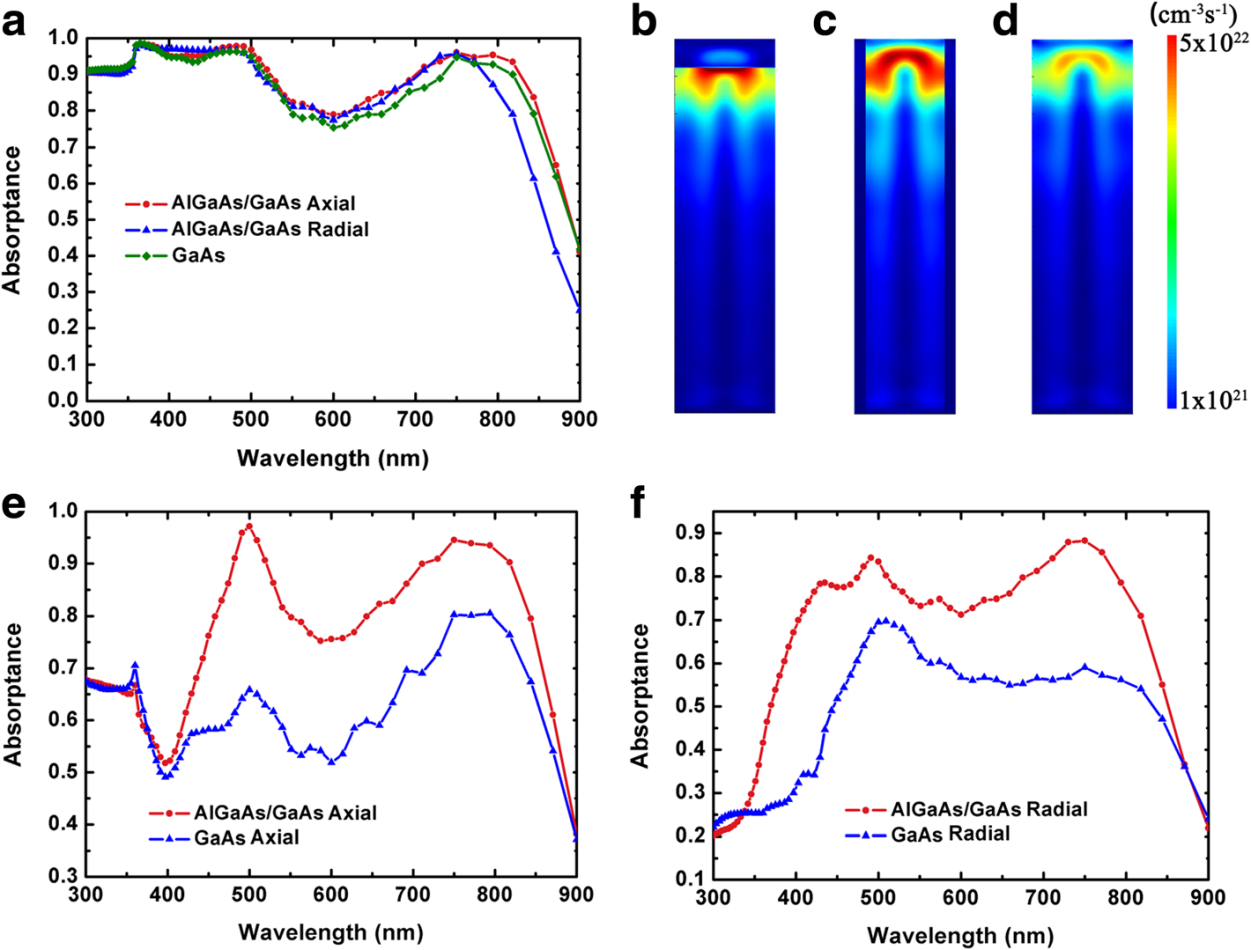
**a** The absorption spectra of the GaAs nanowire and its AlGaAs/GaAs counterparts with axial and radial heterostructures. The vertical cross section of optical generation profiles in the **b** AlGaAs/GaAs axial heterostructure nanowire, **c** AlGaAs/GaAs radial heterostructure nanowire, and **d** GaAs nanowire. **e** The absorption spectra of the intrinsic regions in GaAs nanowire axial pin junction solar cells and its AlGaAs/GaAs counterparts. **f** The absorption spectra of the intrinsic regions in GaAs nanowire radial pin junction solar cells and their AlGaAs/GaAs counterparts

The absorption properties of the AlGaAs/GaAs heterojunction NWs and GaAs NWs are shown in Fig. 2. For axial junction NWs, the lengths of top p-regions and bottom n regions are 150 and 200 nm, respectively. For radial junction NWs, the thickness of p type shells is 20 nm and the radius of the inner n regions is 20 nm. The absorption spectra of the AlGaAs/GaAs and GaAs NWs are almost the same, except that the absorption of AlGaAs/GaAs radial heterojunction NWs drops at wavelengths near GaAs bandgap. At wavelengths around 900 nm, the light propagated in the NWs is concentrated near the side surface, while for the AlGaAs/GaAs radial heterojunction NW, the light propagated in AlGaAs shell cannot be absorbed effectively. Figure 2b–d shows the cross sections of the generation profiles. Due to the lower absorption ability of AlGaAs, only a small fraction of carriers are generated in AlGaAs region; therefore, the recombination loss in the heavily doped AlGaAs region is expected to be not very serious. For AlGaAs/GaAs NWs with axial junctions, most of the optical generation concentrates at the AlGaAs/GaAs interface. While for AlGaAs/GaAs NWs with radial junctions, most of the photocarriers are confined in the GaAs core and blocked away from the NW surface; thus, the surface recombination loss is expected to be suppressed. According to our previous work [15], for NW solar cells with pin junctions, the photo-generated carriers in i-region account for most of the efficiency; therefore, we extract the optical absorption in i-region and calculate the corresponding absorption spectra. For both axial and radial NWs, higher i-region absorption can be achieved in AlGaAs/GaAs heterojunction NWs thanks to the ineffective absorption in p type AlGaAs regions.

**Fig. 3:**
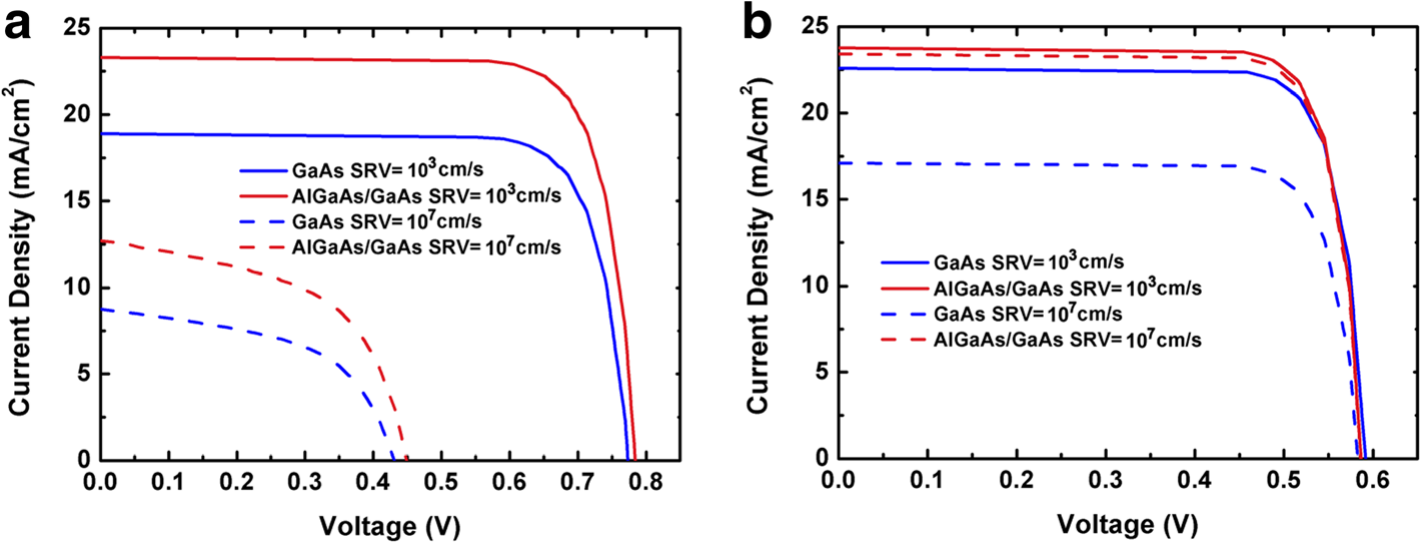
The current–voltage characteristics of the GaAs and AlGaAs/GaAs nanowire **a** axial and **b** radial pin junction solar cells with surface recombination velocities of 10^3^ and 10^7^ cm/s

The optical generation profiles are incorporated into the electrical tool to investigate the potential increase in device conversion efficiency induced by AlGaAs/GaAs heterojunctions. The current–voltage characteristics of the considered devices are calculated and plotted in Fig. 3. Two typical SRVs, 10^3^ and 10^7^ cm/s, are considered during the calculation, corresponding to NW surfaces with and without proper passivation [6, 8, 9]. For axial pin junction NWs with low surface recombiantion, by using AlGaAs for the p top segment instead of GaAs, the conversion efficiency increases from 11.6 to 14.5%. The enhancement of efficiency is mostly attributed from the photo current, which increases from 18.9 to 23.3 mA/cm^2^ at zero bias. Similar phenomenon is observed in radial NWs; the efficiency increases from 10.8 to 11.3% by using AlGaAs/GaAs heterojunctions, with short circuit current increases from 22.6 to 23.8 mA/cm^2^. With high SRV, the performance of axial NWs is dramatically damaged for both AlGaAs/GaAs NWs and GaAs NWs due to the exposed surface of the i-regions. However, the short circuit current enhancement still exists in AlGaAs/GaAs NWs even with a high SRV of 10^7^ cm/s, which comes from the suppressed recombination at the top p-region and the top contact. For AlGaAs/GaAs radial NWs, the efficiency is only slightly affected by the surface recombination thanks to the AlGaAs shell, which confines the photocarriers in the i-region and creates a barrier protecting them from reaching the NW surface. While for GaAs radial NW, the efficiency decreases from 10.8 to 8.05% with SRV increases from 10^3^ to 10^7^ cm/s, and the short circuit current decreases from 22.6 to 17.1 mA/cm^2^.

**Fig. 4:**
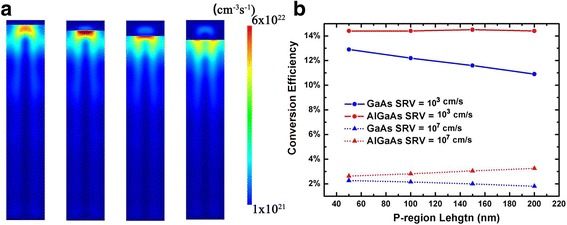
**a** The vertical cross section of optical generation profiles in AlGaAs/GaAs nanowire axial pin heterojunction solar cells with different *p*-region lengths. **b** The conversion efficiency of GaAs and AlGaAs/GaAs nanowire axial solar cells as functions of p-region length

It has been reported that the volume of heavily doped region has great influence on the conversion efficiency, especially for the regions where strong optical generation may occur. In this work, the performance of NWs with various p-region volumes is investigated. In Fig. 4a, the optical generation profiles of axial AlGaAs/GaAs junction NWs with different p-region lengths are plotted. As the p-region length varying from 50 to 200 nm, the optical generation hot spot moves towards the bottom of the NW, and the majority of optical generated carriers are confined below the AlGaAs region. The corresponding conversion efficiencies are calculated as well. The results show that, with low SRV, the increase of p-region length has no obvious influence on the conversion efficiency of AlGaAs/GaAs NWs, although the overall absorption tends to decrease with the increasing AlGaAs volume. What is more, longer AlGaAs region keeps most of the photocarriers further away from the top contact, and fewer minority carriers can be recombined at the contact. However, for GaAs NWs, the conversion efficiency decreases linearly with the increase of p-region length, due to the increasing number of photocarriers generated in top p-region. In the case of the high SRV, the conversion efficiency of AlGaAs/GaAs NWs even increases with the p-region length, because the optical generation in AlGaAs is concentrated at the center of the NW and away from the surfaces, leading to lower surface recombination compared to GaAs regions. From the discussion above, we can conclude that, using AlGaAs for the top p-region instead of GaAs, a relatively long top region can be employed without degenerating device performance. And for NWs with axial junction, a long top region could facilitate the fabrication and contacting of NW array solar cells.

**Fig. 5:**
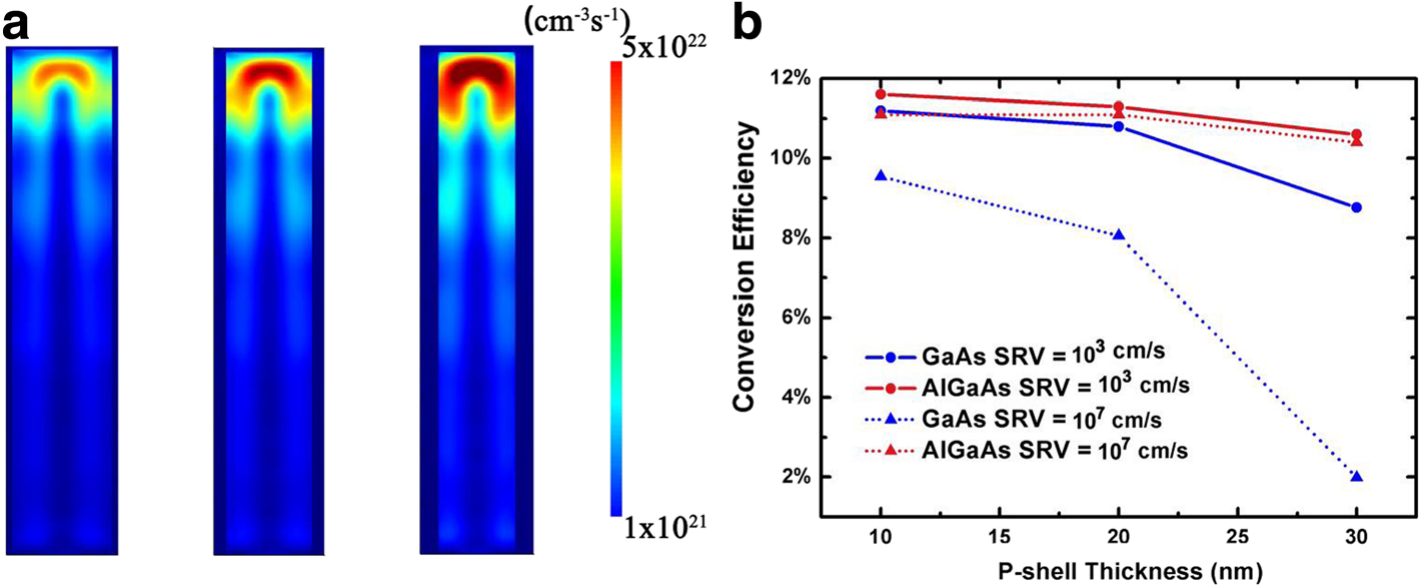
**a** The vertical cross section of optical generation profiles in AlGaAs/GaAs nanowire radial pin heterojunction solar cells with different *p*-shell thicknesses. **b** The conversion efficiency of GaAs and AlGaAs/GaAs nanowire radial solar cells as functions of p-shell thickness

The performance of radial NWs with different p-shell thicknesses has also been calculated. Figure 5a shows the optical generation profiles of the AlGaAs/GaAs radial NWs. Similar with that in axial NWs, the majority of photocarriers are generated in GaAs. The conversion efficiency of both AlGaAs/GaAs and GaAs NWs decreases with the increasing p-shell thickness. In the case of low SRV of 10^3^ cm/s, the effect of surface recombination is almost negligible; thus, the efficiency degeneration mainly comes from the increasing number of photocarriers generated in the p-shell. However, AlGaAs/GaAs NWs show better tolerance to p-shell thickness, as most of the optical generation can be confined in inner GaAs regions. With the SRV increases from 10^3^ to 10^7^ cm/s, the conversion efficiency of AlGaAs/GaAs NWs is only slightly decreased, as the photocarriers are protected by AlGaAs shells from the surface. And for NWs with thicker AlGaAs shells, as fewer carriers can reach and recombine at the surface, the device performance is less degenerated. On the contrary, the performance of GaAs NWs is seriously damaged by high surface recombination, especially in the cases of thick p-shells. Because for GaAs radial NWs, the photocarriers generated in the p-shell can be recombined easily at the surfaces. With a p-shell thickness of 30 nm, the conversion efficiency of GaAs NWs is only 1.98%, while the corresponding AlGaAs/GaAs NWs show an efficiency of 10.4%, 8.42% higher than that of the GaAs NWs.

## Conclusions

In this work, we use a coupled 3-D optoelectronic simulation to investigate the performance of AlGaAs/GaAs and GaAs NW pin heterojunction array solar cells. Compared with GaAs NWs, AlGaAs/GaAs NWs can confine most of the optical generation in the active regions, reducing the recombination loss exists in the heavily doped regions, and form barriers for minority carriers, protecting them from surface or contact recombination. For AlGaAs/GaAs axial NWs, by using AlGaAs for the top p-region instead of GaAs, we can allow a relatively long top region without degenerating device performance, which could facilitate the fabrication and contacting of NW solar cells. And for radial NWs, the efficiency of AlGaAs/GaAs NWs can be maintained at a relatively high value with very high surface recombination. From this study, we can conclude that employing AlGaAs/GaAs heterojunctions is an effective and practical method to enhance the performance of GaAs NW solar cells.

## Abbreviations

3D: Three-dimensional
NW: Nanowire
SRH: Shockley-Reed-Hall
SRV: Surface recombination velocity
